# An opportunity to sleep well in hospital: development of a multi-level intervention to improve inpatient sleep (ASLEEP) using behaviour change theories

**DOI:** 10.1186/s40359-024-02281-9

**Published:** 2024-12-27

**Authors:** Anna Louise Hurley-Wallace, Wendy Bertram, Emma Johnson, Vikki Wylde, Katie Whale

**Affiliations:** 1 NIHR Bristol Biomedical Research Centre, University Hospitals Bristol and Weston NHS Foundation Trust and University of Bristol, Bristol, UK; 2 Musculoskeletal Research Unit, Bristol Medical School, University of Bristol, Bristol, UK

**Keywords:** Inpatient Sleep, Orthopaedic inpatients, Acute Medicine, Hospital inpatients, Intervention development, Behaviour Change, Sleep Behaviour

## Abstract

**Background:**

Sleep is substantial issue for hospital inpatients and can negatively affect healing and recovery. There is a good evidence-base for interventions which can improve sleep, however currently they are not being implemented into NHS practice. To address the evidence-practice gap, we have conducted early-phase development for an inpatient sleep intervention (ASLEEP); a multi-level intervention to improve inpatient sleep in UK hospital wards.

**Methods:**

We used an iterative development process incorporating Patient and Public Involvement and Engagement, ward staff surveys and stakeholder consultations (orthopaedic and acute medicine), and theoretical mapping using behaviour change theories. Development took place in four stages: identification of existing patient-level intervention components to improve sleep in hospital; identification of environmental barriers and facilitators to sleep in hospital; consultation with health professional stakeholders; and final theoretical mapping using the COM-B model and Theoretical Domains Framework, also considering who holds ‘change power’ for each change construct.

**Results:**

We identified 18 variables contributing to inpatient sleep, which are malleable to change universally across hospital wards. Central domains for change were identified as the ward environment context and resources; to reduce noise from equipment (material resources), and social influence; to modulate staff and patient noise awareness and behaviours (group norms). Change power mapping identified key stakeholders as patients, ward staff, procurement/estates, and NHS management.

**Conclusions:**

Improving sleep in hospital requires a whole-systems approach which targets environmental factors, staff behaviour, and patient behaviour. We have provided recommendations for a multi-level intervention, highlighting core areas for change and essential stakeholders who must be involved to progress implementation. The next stage of development will involve operationalising recommendations and piloting, including evaluating mechanisms of change. It will be important to continue working with a broad range of stakeholders to bridge the evidence-practice gap and support sustainable practice adoption.

**Supplementary Information:**

The online version contains supplementary material available at 10.1186/s40359-024-02281-9.

## Background

Everyday approximately 115,000 people in the UK stay overnight in hospital [1]. Sleep disturbance in hospital is a substantial problem with approximately 50–70% of inpatients reporting reduced sleep quality and duration [2]. The ward environment is a central barrier to good sleep with noise, lighting, and nursing care highlighted as affecting sleep quality [3–5].

Poor sleep can delay recovery and extend hospital stays by 1–8 days, with significant NHS cost implications [6]. Shortening hospital stay is an NHS priority, with sleep deprivation highlighted as important [7]. A government green paper further emphasised that guidance to improve sleep in hospitals requires review and update [8]. However, no research has yet addressed this or implemented evidence-based changes.

Sleep directly impacts wound healing and immune function [9, 10]. Reduced sleep increases the risk of post-surgical complications, including delirium [11, 12], and can lower memory function, energy, and strength [13]. Reduced sleep increases pain sensitivity by activating proinflammatory markers [14–16], where pre-operative sleep disturbances are associated with increased post-operative pain severity [17].

Undergoing surgery is a common reason for staying overnight in hospital. Total knee and hip replacements are the second and third most common elective surgical procedures in UK, with approximately 200,000 annually [18]. Most patients spend 1–3 nights in hospital after surgery [19, 20]. Recovery programmes aim to reduce recovery time and length of stay by controlling pain and supporting mobilisation [21]. Nonetheless, poor sleep can be a barrier to timely recovery and discharge through delayed healing, increased pain, and increased risk of post-surgical complications [9, 11, 17, 22].

There is strong evidence that existing interventions can improve inpatient sleep. Patient-level interventions targeting noise, light, and patient anxiety have shown effectiveness, based on meta-analysis results incorporating data from 5375 patients [23]. Environment-focused interventions, such as reducing night-time care interruptions, also show promise [3, 24].

Despite existing evidence, there is an evidence-practice gap in integrating sleep interventions into NHS practice. Hospital contexts are challenging for the development of sleep interventions, as the physical and sociocultural environment, including existing policies, require consideration [25]. Research indicates that ward staff are supportive of improving sleep, but lack knowledge and guidance for making changes [4, 26]. To successfully integrate evidence-based changes to improve sleep within NHS practice, research must identify barriers and facilitators to change, and work with NHS stakeholders to address issues.

For multi-level interventions which require engagement from various stakeholders (e.g., policymakers, staff, patients), behaviour change theories offer valuable insight into how to enact successful, sustainable change within complex systems. This study conducted the first stage of work to bring together evidence-based approaches for improving inpatient sleep, into a new intervention package; the ASLEEP intervention. This included exploring patient views of sleep interventions, health professional views of the barriers and facilitators to inpatient sleep, and organising evidence using theoretical mapping.

## Methods

Ethical approval was given by the University of Bristol Faculty of Health Sciences Ethics Committee on 13/07/2022 (reference 10490). Informed consent to participate was obtained from all research participants in the study.

This report follows the Good Reporting of A Mixed Methods Study guidance [27] (Additional File 1), comprising four phases:

1. Understanding patient views of existing intervention approaches (systematic review, Patient and Public Involvement and Engagement [PPIE]).
2. Understanding ward sleep environments and practices, including identification of targets for change (staff survey, concept map, behaviour change theoretical mapping).
3. Stakeholder consultation (follow-up staff survey, consultation with NHS staff).
4. Theoretical mapping of change factors (iterated theoretical map, identification of target variables and malleability to change).

The methods and results for each phase are presented together to aid understanding.

### Phase 1: Patient-level intervention approaches

Objectives: To gain feedback from a PPIE group on the acceptability of existing interventions for improving inpatient sleep, how to implement them within the NHS, and any potential barriers.

#### Methods

We previously conducted a systematic review and meta-analysis of trials of inpatient sleep interventions [23]. Results were discussed with a PPIE group comprising five patient-partners with recent experience of overnight hospital stays. Three were recruited using an advert circulated by People in Health (West of England). Two were recruited through existing PPIE groups.

PPIE group members had experience of orthopaedic surgery (total knee replacement *n* = 2, flat foot reconstruction *n* = 1), gall bladder removal (*n* = 1) and gynaecological surgery (*n* = 1). Length of stay ranged from 1 to 10 nights. Time since surgery at the point of the PPIE meeting ranged from 1 to 8 months.

The group met once for a 2-hour Zoom meeting in October 2022, facilitated by the research team (EJ, WB, KW). The aim of the meeting was to review the findings of our previous meta-analysis of sleep interventions, and explore acceptability and preferences for the sleep intervention approaches. The meta-analysis [23] identified six interventions that were effective at improving inpatient sleep:

- Relaxation techniques.
- Physical sleep aids (ear plugs and eye mask).
- Music.
- Counselling.
- Massage and acupuncture.
- Environment changes (light, noise, observation, monitoring).

The group were first asked about their experiences of overnight stays in hospital and what helped or prevented them from sleeping. They were then shown a slide of the six intervention areas and asked the following questions:

- What are the main barriers for a study on improving sleep in hospital?
- Would anything stop you from taking part?
- How could we test if these approaches work at:
  - Individual-level.
  - Ward-level.
  - Hospital-level.
- How should we measure changes?

#### Results

The PPIE group supported improving inpatient sleep and had all experienced sleep disruption. They saw benefit in all approaches, but felt that counselling and massage may not be suitable for everyone, and possibly difficult to access due to needing trained staff and a suitable location for the massage treatments. The two most acceptable approaches were physical sleep aids and relaxation because they could be easily provided and used individually as desired.

The group additionally highlighted that worry and anxiety about being in an unfamiliar environment as important. They suggested a leaflet could be sent to patients to help them prepare for their stay including what to expect on the ward, and a list of recommended items to enhance comfort (e.g., earphones, pillow spray). For unplanned stays, a leaflet could be given on admission with information and advice for what their family or friends could bring for them.

### Phase 2: Ward environment-level factors

Objectives: To understand the sleep environment and sleep-related practices on NHS hospital wards, and identify target areas for change.

#### Methods

Orthopaedic ward staff from 19 UK hospitals were invited to take part in a survey. This included tertiary referral and general district sites treating urban, rural and diverse demographic populations. Hospital sites were identified by the research team using existing contacts. The survey was distributed online (JISC surveys) and on paper between 22 July 2022 and 18 March 2023.

The survey was designed specifically for the study and included four sections, comprising a mixture of open and closed questions. These were: [1] ‘About your hospital’ (ward environment and structure, shift patterns, prescribing roles) [2], ‘Care after surgery’ (sleep-related care routines and procedures) [3], ‘The ward environment and sleep’ (environment at night, set routines, night-time changes and current policies supporting sleep, available sleep aids and medications, staff views on the importance of patient sleep), and [4] ‘Improving inpatient sleep’ (barriers and facilitators, other factors not mentioned). Staff demographic data was also collected.

Results were anonymised. Qualitative responses were condensed into a concept map of ward environment-level barriers and facilitators. Quantitative data summaries (frequency counts) were conducted in Microsoft Excel. Relevant theories from the health behaviour change literature were then considered for theoretical mapping of the results. This was done to help identify target areas for change.

#### Results

Thirty-seven responses were received from three hospitals. Participants included nurses (*n* = 20), healthcare assistants (*n* = 8), nurse sisters (*n* = 4), trainee nurse associates (*n* = 2), clinical nurse managers (*n* = 2) and a consultant orthopaedic surgeon (*n* = 1).

Wards had a mixture of open bays (4–5 patients/ up to 12 patients) and individual rooms. A regular night shift pattern was noted from 7pm to 7.30am.

Post-operative care routines depended on surgery type, patient condition, and time of return from theatre. Night-time observations were guided by patients’ Early Warning Score (EWS). Post-op day 1 observations were 4-hourly, post-op day 2 onwards observations were every 4–6 h. Comments indicated that patients in bays are often woken by other patients being attended to.

There was a consensus on sleep times; lights dimmed by 11pm, turned back up between 6am-7am, with low levels of noise encouraged at night. Nonetheless, there were mentions of loud call bells, banging doors, and poor awareness of noise levels by colleagues. A small number of responses indicated use of a call bell ‘night mode’ function. Some staff stated their ward is ‘quiet and calm’ with hot drinks provided in the evening to help patients settle.

Nineteen staff indicated there was a strategy or policy in place to improve inpatient sleep. Most responses (*n* = 25) indicated sleep aids (eye masks and ear plugs) were available on request. One ward had discontinued sleep aids, one offered sleep aids to every patient. Prescribing sleep medication was described as ‘not routine’.

Results indicated good knowledge of the impact of sleep on patients’ recovery and wellbeing. Staff highlighted poor sleep as affecting patients’ pain levels (*n* = 14), participation in care (*n* = 8), general wellbeing (*n* = 8), mental health (*n* = 7), recovery (*n* = 7), and mood (*n* = 5).

##### Identification of barriers and facilitators to inpatient sleep

**Fig. 1:**
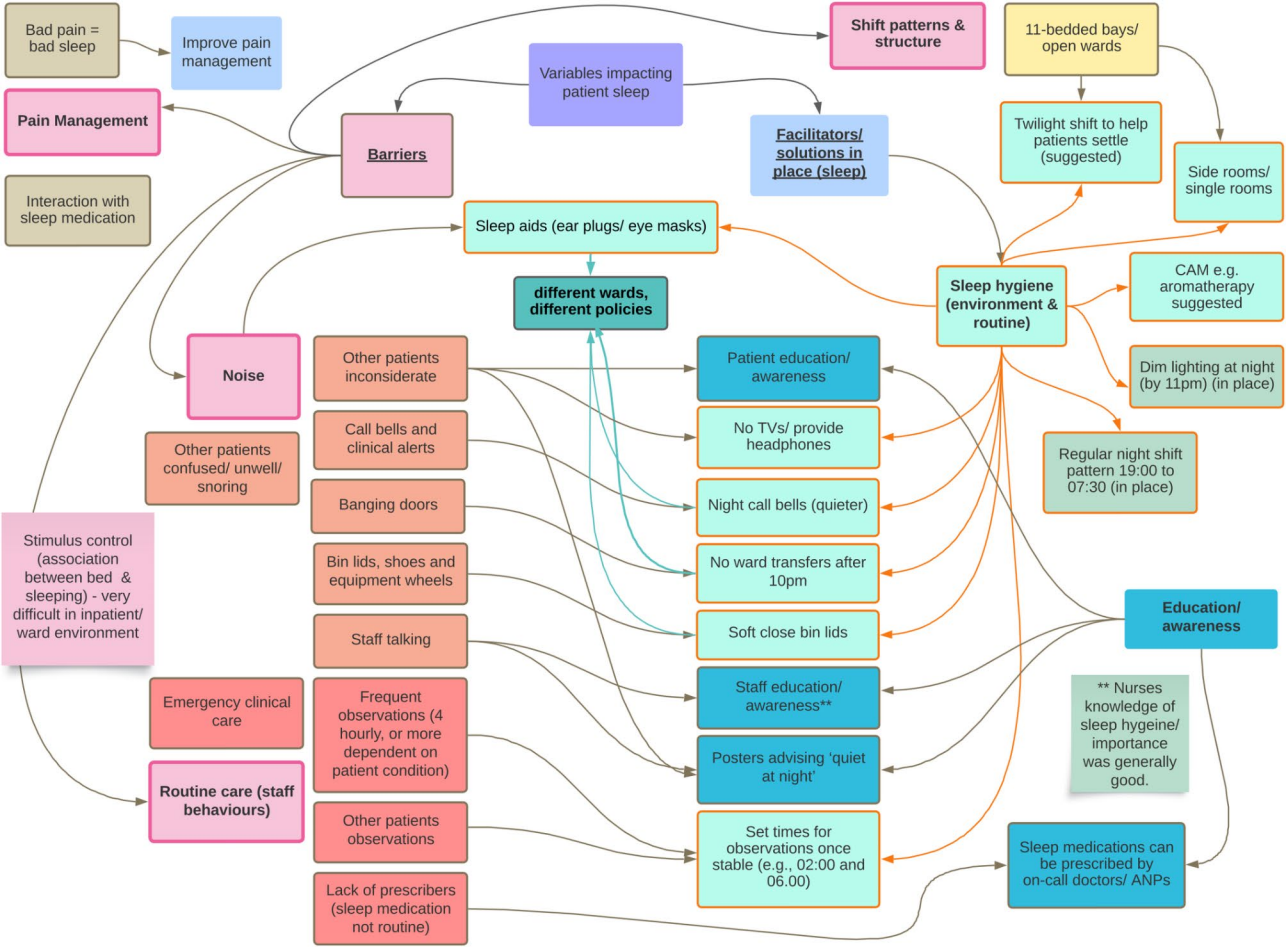
A concept map of ward environment-level barriers and facilitators impacting inpatient sleep

Survey questions 28 and 29 asked staff to identify barriers and facilitators to good inpatient sleep. Qualitative responses were visualised as concept map. (Fig. 1).

#### Theoretical mapping using behaviour change theories

Environment-level variables identified in the concept map were mapped to behaviour change theories. Application of theory is important for understanding what changes are needed and how change can be achieved [28].

Theoretical mapping was undertaken using the COM-B model [34] and Theoretical Domains Framework (TDF) [29]. These theories were chosen as they provide a comprehensive list of environmental constructs and overarching domains or areas that require change. Variables affecting inpatient sleep were initially conceptualised as COM-B physical and social opportunity factors, which were then mapped to TDF domains; environmental context and resources, and social influence (Table 1).

**Table 1:** Theoretical mapping of variables affecting sleep

COM-B component	TDF domain	Variable
Physical opportunity	Environmental context and resources	Lighting
		Doors
		Bins
		Call bells
		Medical equipment
		Care routines
		Shift patterns
		Critical medical and hospital-wide incidents
Social opportunity	Social influence	Staff talking (social awareness)
		Staff talking (patient care)
		Staff/ ward radio
		Patients talking
		Patients’ TV or devices

### Phase 3: Stakeholder consultation

Objectives: To consult staff stakeholders on variables identified in Phase 3, understand feasibility of ward environment changes, and identify barriers to change.

#### Methods

Two approaches were used to gain stakeholder views: (i) stakeholder meetings with NHS staff, and (ii) follow-up ward staff survey.

##### Stakeholder meetings

Consultations were carried out with Consultants (orthopaedics, respiratory), Senior Ward Nurses (orthopaedics, respiratory, acute medicine), a Ward Matron, and Ward Administrator. The consultations were facilitated by one researcher, KW. One meeting took place using videoconference (orthopaedic consultant), all other meetings took place face-to-face at the hospital site. Stakeholders were provided with a summary of findings from the first staff survey and the variables identified from the theoretical mapping. Discussion questions were:

- What variables would you want to change in an ideal world?
- What can be feasibly changed within the ward environment (physical structure procedural/ care delivery change)?
- What timepoint(s) are best for sleep packs to be given to participants (pre-op, admission, both)?
- Any other physical or procedural issues identifiable?

Stakeholders were additionally asked about key contacts or other job roles that should be included in consultation.

Initial stakeholder meetings identified that the key variables affecting sleep were universal across wards, thus intervention development could be expanded to other wards and patient groups in addition to orthopaedics to provide maximum patient benefit. Following this, respiratory and acute medicine staff were invited for consultation, and to participate in the follow-up survey.

##### Staff follow-up survey

The follow-up survey was distributed online or on paper between 6 May 2023 and 17 August 2023. Eight staff from the Phase 2 survey who consented to be contacted were emailed an online survey link. The paper version of the survey was distributed to orthopaedic and acute medicine wards at one South West NHS site, using opportunity sampling (the researcher delivered paper questionnaires directly to the ward receptions). No contact details were collected. Surveys were anonymous.

The survey presented variables identified in phase 2 (see Table 1), which were split into two categories: environment and equipment (e.g. doors, lighting, call bells, bins), and other noise (e.g. talking, clinical care, ward radio, patient devices). For each variable, staff were asked if they would change this in an ideal world (yes/no), and the ease of change (easy/ possible/ too difficult), with open-ended responses on each category (‘What makes this change easy/ difficult?’ and ‘What support is needed to make this change?’). Two open-ended questions asked staff whether they would feel comfortable asking patients to turn off devices or use headphones, and for any other barriers.

Blank responses to the ‘ideal change’ and ‘easy to change’ questions were coded as ‘no’ or ‘too difficult’ for data entry. Analyses were conducted in Microsoft Excel.

#### Results

##### Stakeholder consultation

Stakeholders were highly supportive of work to improve inpatient sleep and recognised this as an important issue. Views on the causes of poor sleep matched the results from Phase 2. Discussion of care routines and night-time observations reiterated use of the EWS. It was noted that if patients were stable it could be possible to reduce overnight observations between the hours of 12am-6am, however changes to the recommendations would require review to ensure clinical safety. To address staff talking, noise monitors that provide visual signals of noise levels e.g., SoundEar II [30] were suggested to improve awareness.

In addition to universal ward variables, care aspects affecting specific patients groups were discussed e.g., oxygen masks for respiratory patients, foot/calf pumps in orthopaedics. To address this, it was suggested that ‘bolt-on’ modules could be used to augment the core recommendations.

##### Ward staff follow-up survey

Thirty-eight staff completed the survey (two online, 36 paper). Respondents were from acute medicine wards (acute care of the elderly; *n* = 17, sub-acute care/respiratory; *n* = 18), and orthopaedic wards (*n* = 3).

**Fig. 2:**
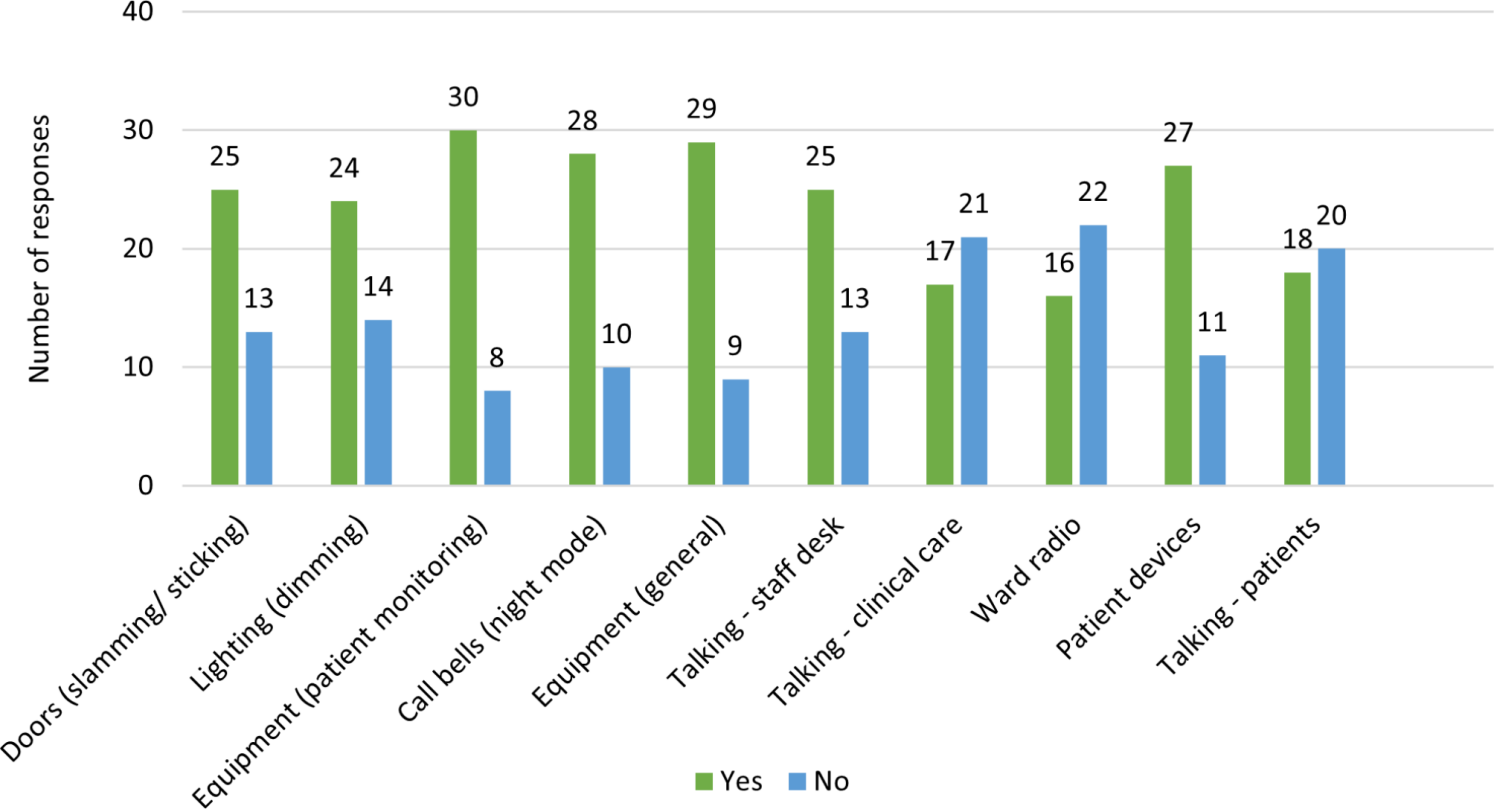
Staff responses to ‘In an ideal world, would you change…?’ for all sleep-related ward environment variables

The ‘ideal change’ results are presented in Fig. 2. Staff agreed that equipment and the structural environment (e.g., doors and lighting) should ideally be changed, and staff social talking should be reduced. Comments indicated that some colleagues are ‘unaware’ of their volume.

**Fig. 3:**
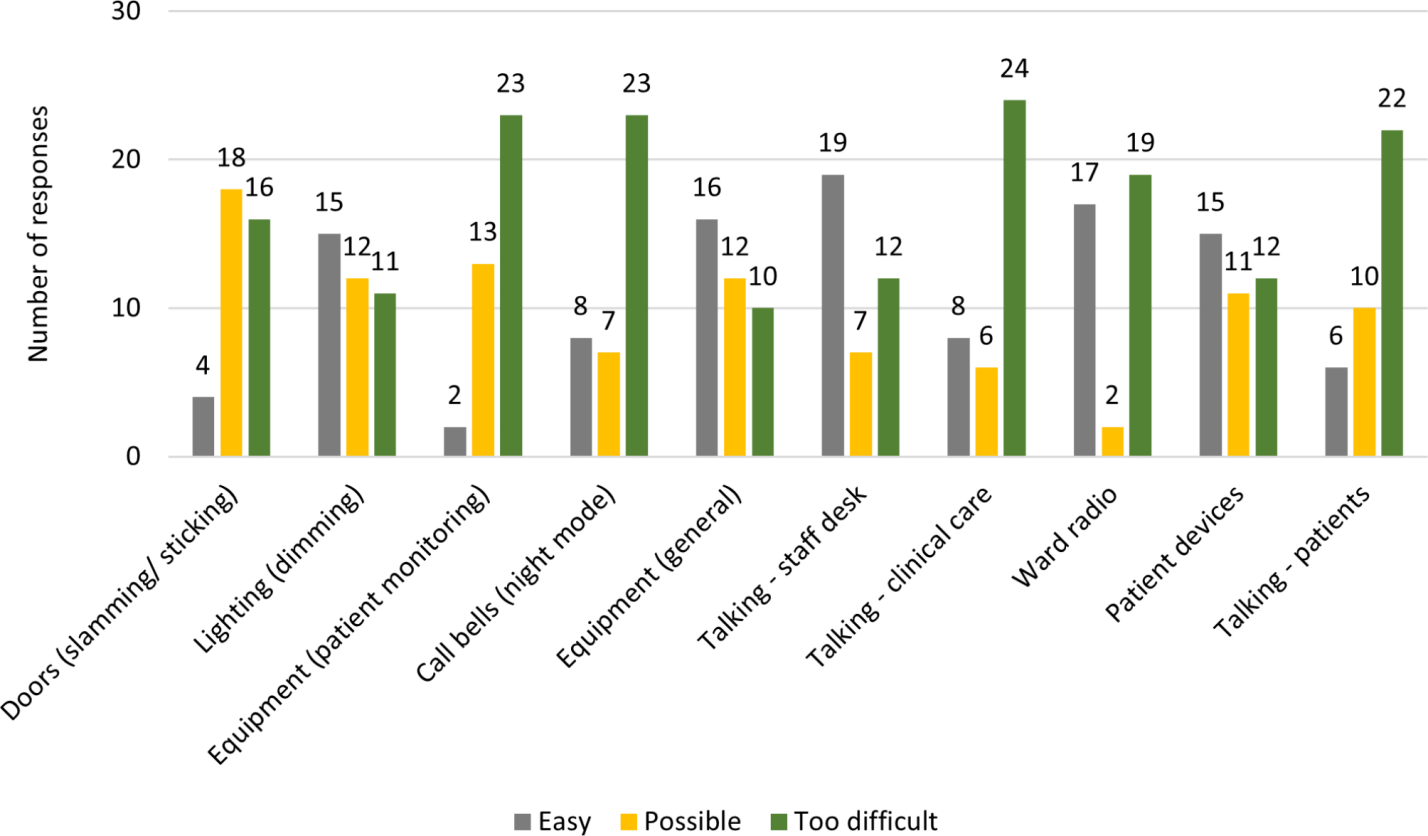
Staff responses to ‘How easy is … to change?’ for all sleep-related ward environment variables

General equipment was viewed as easy to change, with several suggestions of soft close bin lids. Equipment reported as ‘ideally would change but too difficult’ included clinical monitoring equipment and call bells (Fig. 3). Responses indicated that some staff were concerned about patient safety if the volume was lowered, and alerts went ‘unnoticed.’

Noise from clinical care conversations was reported as too difficult to change. Responses emphasised that patients could be confused, uncooperative, and hard of hearing, thus increasing the volume of talking during interactions. Social staff talking and patient devices were most frequently marked as easy to change, with comments indicating that simple reminders and providing headphones could resolve the issues.

Support needed to implement changes included all staff being ‘proactive’ and ‘cooperative’, with recommendation for a change management strategy. Three respondents suggested staff education would be beneficial.

Twenty-nine staff reported they would be comfortable approaching patients to turn off devices or use headphones (‘yes’=25, ‘comfortable to approach’=4). Three participants indicated they would not be comfortable to approach patients, by responding ‘no’ or ‘not always’. Alternative approaches to address patient device use were offered, for example asking patients to ‘turn [the volume] down, but not off.’ Respondents noted that using headphones presents difficulties for hearing impaired patients.

Additional barriers to sleep were highlighted as room temperature (*n* = 2), patient transfers (*n* = 3), sending patients for tests (CT, X-ray etc.) (*n* = 4), confused patients being noisy (*n* = 5), uncomfortable mattresses (*n* = 1), and late administration of medications (*n* = 1).

### Phase 4: Mapping change factors to improve inpatient sleep

Objectives: To integrate phase 3 results with the theoretical mapping, identify variables malleable to change, and identify ‘change power’ for each construct.

#### Methods

Phase 3 results were added to the theoretical mapping table. A column was added to reflect the malleability of each variable and identify which variables could be targeted for change within the NHS. A ‘change power’ column was added to identify the stakeholders needed to implement changes. For example, new ward equipment requires support from NHS management and estates/procurement.

#### Results

Final theoretical mapping is presented in Table 2. Twenty-one variables impacting inpatient sleep were identified, mapping to five COM-B areas and 10 TDF constructs, with 18 variables malleable or partially malleable for change.

**Table 2:** Final theoretical mapping of variables impacting inpatient sleep. New variables are indicated with *

COM-B component	TDF domain	TDF construct	Change power	Variable	Malleable for change
Physical opportunity	Environmental context and resources	Material resources	- Management & leadership - Estates/ procurement	Lighting	Yes
				Doors	Yes
				Bins	Yes
				Call bells	Yes
				Medical equipment	Partially
		Organisational culture and climate	- Management & leadership	Care routines	Partially
				Shift patterns	No
				Patient tests and transfers*	No
		Salient events /critical incidents	- Management & leadership - Ward staff	Critical medical and hospital-wide incidents	No
Social opportunity	Social influence	Group norms	- Ward staff - Patients	Staff talking (social awareness)	Yes
				Staff talking (patient care)	Yes
				Staff/ward radio	Yes
				Patients talking	Yes
				Patients’ TV or devices	Yes
Psychological capability	Knowledge	Procedural knowledge (knowing what to do)	- Ward staff - Patients	Staff knowledge of night-time protocols*	Yes
				Patients’ knowledge of night-time protocols*	Yes
		Knowledge of task environment	- Patients	Patients’ knowledge of ward environment (what to expect) *	Yes
Automatic motivation	Reinforcement	Consequents	- Patients	Patient awareness of consequences (social or physical) of not following night-time protocols*	Yes

### Proposed ASLEEP intervention

We identified 18 variables impacting inpatient sleep which can be targeted. Considering all stakeholder input throughout the development process, we interpret that the core domains for change relate to the ‘opportunity’ to sleep well in the hospital environment. Specifically, ward environment context and resources; to reduce noise from equipment (material resources), and social influence; to modulate staff and patient noise awareness and behaviours (group norms), should be a priority within the intervention. Table 2 additionally proposes domains for change relating to ‘capability’ and ‘motivation’, which more broadly cover the psychological and motivational processes underpinning why procedures and protocols that impact night-time noise levels are not aways adhered to.

Based on these findings, the ASLEEP intervention recommendations are:

1. Environment and equipment:
  Lighting dimmed at night.
  Ward equipment to be modified or replaced with low noise options (e.g., soft close bins).
  Medical equipment used with night mode if available (e.g., call bells).

2. Staff awareness and behaviour:
  Staff noise reduction (non-clinical conversations).
  No electronic device noise between 11pm-6am. (e.g., ward radio).

3. Patient awareness and behaviour:
  Provide patient sleep packs, including sleep aids, relaxation resources and information leaflets.
  Patient noise reduction and a ‘quiet time’ policy between 11pm-6am.

Consultations identified that ‘bolt-on’ recommendations for specific patient groups could be used to augment the universal recommendations. This could include sleep aids suitable for use with specialist equipment e.g., neck pillows for patients using oxygen masks.

## Discussion

This early-phase intervention development work has benefitted from a combined theory- and systems-based research perspective [28], which helped break down the complex issue of inpatient sleep into areas of change and specific variables that are targetable within an NHS context. This study supports that a whole-systems approach is needed to implement evidence-based approaches to improving inpatient sleep [31].

Insights from NHS patient and staff stakeholders identified key target areas for change at patient, ward, and hospital-level, which must be addressed synchronously. We have identified that reducing noise levels is pivotal to giving patients the opportunity sleep well. To achieve this, several material resource variables require change, including equipment and ward structure (e.g. doors), as well as staff and patient group norms that contribute to noise levels, such as talking and device use. This reflects findings from previous studies, which all highlight noise as a central issue [3, 4].

A major challenge this work has identified is bridging the gap between research evidence on sleep in hospitals and real-world implementation [32]. Medical Research Council (MRC) guidance highlights the importance of working with a range of stakeholders throughout intervention development, including those whose personal or professional interests are affected, to enhance potential for real-world implementation [28]. In addition to conducting consultation with patient and staff stakeholders, this study sought to bridge the evidence-practice gap by considering the wider hospital context and identifying other stakeholders who hold ‘change power’. By adding change power to the theoretical map, we identified that the people who hold responsibility for factors impacting sleep in hospitals differs between constructs, with additional stakeholders identified as domestic staff, estates and procurement, and NHS management. Further work to develop ASLEEP will actively seek to involve these broader stakeholders in consultations.

Stakeholder consultation undertaken in this study also highlighted that ASLEEP could benefit patient groups beyond orthopaedics, as the domains and constructs impacting inpatient sleep are universal across wards. Good sleep is essential to wound healing, reduces the risk of post-surgical complications [9, 12], and can reduce pain [14]. Whilst pertinent to orthopaedic surgical recovery [21], patients in acute wards can equally benefit from improved sleep. Poor sleep in acute hospital settings is known to increase pain, reduce strength, and adversely affect respiratory function [33].

This development work took a combined theory- and systems-based research perspective [28], which has been useful for breaking down a complex issue into areas of change that are targetable within an intervention. This breakdown has been equally useful for presenting target areas for change to stakeholders throughout consultations. The TDF [29] has been important in understanding and explaining environmental factors to stakeholders, and we have mapped the TDF to the COM-B [29, 34] to present a holistic view of how inpatient sleep can be tackled through organisational, group, and individual behaviour change.

The next step in ASLEEP development is operationalisation into a useable toolkit, and pilot testing in NHS orthopaedic and acute hospital ward settings to ensure the intervention is acceptable and sustainable. To enhance potential for real-world implementation, further stakeholder work using knowledge mobilisation approaches is recommended [35]. This involves seeking consultation from broader stakeholders that hold power to enact change. At a hospital-level, this study has identified domestic staff, estates, procurement and NHS management as important. Additionally, this broader stakeholder group should include policymakers and commissioners. Whilst there is little practical guidance on how to onboard high-level stakeholders [36], they can provide valuable insight on how best to operationalise intervention recommendations, and help develop a sustainable practice adoption plan.

At intervention piloting stage, a process evaluation will be needed to understand mechanisms of change or ‘how’ the intervention works, as well as any other contextual or moderating factors that have not yet been identified [28, 37]. Some variables identified within the current study represent moderators, such as hospital-wide bed shortage. These factors are marked as not malleable to change, however, should be considered as moderators when conducting a process evaluation.

In summary, the current work has identified an extensive list of target variables that are malleable to change in an NHS context, through sequential integration of stakeholder input and iterative organisation of findings using behaviour change theories. These targets for change provide a foundation to operationalise and refine ASLEEP as a toolkit that can be flexibly used to improve inpatient sleep in UK hospital wards.

### Strengths and limitations

This work used behaviour change theories to inform early-stage intervention development, and is strengthened by the range of stakeholders involved. Findings help bridge the gap between sleep research and hospital practice by highlighting the variety of people and processes that are necessary to ignite change at multiple system levels, with the mutual goal of improving inpatient sleep. A further strength is the flexible approach to development. As a result of initial stakeholder consultation, this research was quickly adapted during Phase 4 to include input from acute care clinicians. Rapid, adaptive approaches are supported by the intervention development literature [38], with emphasis on centrality of stakeholder input throughout development. A limitation of rapidly adapting is that clinician role details were not collected from acute care staff. Nonetheless, this broadened the potential reach of the intervention in practice.

## Conclusion

We have provided recommendations for ASLEEP, a multi-level intervention to tackle the complex issue of poor sleep experienced by hospital inpatients. These intervention recommendations could benefit patients across hospital wards, including orthopaedic and acute care. Improving sleep in hospital requires a whole-systems approach which targets environmental factors, staff behaviour, and patient behaviour. This work has provided an applied example of how behaviour change theories can be used in early-stage intervention development to breakdown complex healthcare issues. Theoretical mapping has helped identify core areas for change and key stakeholders who should be engaged to progress implementation, including patients, hospital staff, and NHS management. The next stage of development will involve operationalising recommendations and piloting, including evaluating mechanisms of change. It will be important to continue working with stakeholders, and broader policymakers, to bridge the evidence-practice gap and develop a robust service adoption plan.

## Electronic supplementary material

Below is the link to the electronic supplementary material.

Additional File 1: GRAMMS-ASLEEP – Reporting checklist for Mixed Methods

## Data Availability

Data are available on request. Participants were asked on the consent form if they were willing for their information to be shared anonymously to support other research in the future. Anonymised data will be stored on the University of Bristol Research Data Storage Facility (https://data.bris.ac.uk) and will be shared via the University of Bristol Research Data Repository.

## Abbreviations

ASLEEP: Inpatient Sleep Intervention
NHS: National Health Service
UK: United Kingdom
PPIE: Patient and Public Involvement and Engagement
TDF: Theoretical Domains Framework
MRC: Medical Research Council
