# Supplementary material for: An opportunity to sleep well in hospital: development of a multi-level intervention to improve inpatient sleep (ASLEEP) using behaviour change theories
Source: BMC Psychol. 2024 Dec 27;12:788. doi: 10.1186/s40359-024-02281-9 (PMC11681736; doi:10.1186/s40359-024-02281-9)
Supplement: Supplementary file 1 — Additional File 1: GRAMMS-ASLEEP – Reporting checklist for Mixed Methods [file 40359_2024_2281_MOESM1_ESM.docx]

Good Reporting of A Mixed Methods Study (GRAMMS) – checklist^[[1]](#endnote-1)^

Article title: *An opportunity to sleep well in hospital: Development of a multi-level intervention to improve inpatient sleep (ASLEEP)*

| **Item** | Consideration | Comment |
| --- | --- | --- |
| **(1)** | Describe the justification for using a mixed methods approach to the research question | Intervention development study using iterative survey and consultation methods, alongside theoretical integration. |
| **(2)** | Describe the design in terms of the purpose, priority and sequence of methods | Sequence of methods/ phases given at the start of article. |
| **(3)** | Describe each method in terms of sampling, data collection and analysis | Studies and phases are separated, presenting methods and results for each of the surveys and consultations in chronological sequence |
| **(4)** | Describe where integration has occurred, how it has occurred and who has participated in it | Described in methods, iterative intervention development. |
| **(5)** | Describe any limitation of one method associated with the present of the other method | See discussion, strengths and limitations. Included in discussion that methods were rapidly adapted as project progressed. |
| **(6)** | Describe any insights gained from mixing or integrating methods | See discussion, strengths and limitations. Different methods used as appropriate to different groups of stakeholders. |

1. O'Cathain, A., Murphy, E., & Nicholl, J. (2008). The quality of mixed methods studies in health services research. Journal of health services research & policy, 13(2), 92–98. https://doi.org/10.1258/jhsrp.2007.007074 [↑](#endnote-ref-1)
